# Stopping power beyond the adiabatic approximation

**DOI:** 10.1038/s41598-017-02780-3

**Published:** 2017-06-01

**Authors:** M. Caro, A. A. Correa, E. Artacho, A. Caro

**Affiliations:** 1 Department of Mechanical Engineering, Virginia Polytechnic Institute and State University, Falls Church, VA 22043 USA; 2 Quantum Simulations Group, Lawrence Livermore National Laboratory, Livermore, CA 94550 USA; 3 CIC Nanogune and DIPC, Tolosa Hiribidea, 20018 San Sebastián, Spain; 4 Basque Foundation for Science Ikerbasque, 48013 Bilbao, Spain; 5 Theory of Condensed Matter, Cavendish Laboratory, University of Cambridge, Cambridge, CB3 0HE UK; 6 Materials Science and Technology Division, Los Alamos National Laboratory, Los Alamos, NM 87545 USA

## Abstract

Energetic ions traveling in solids deposit energy in a variety of ways, being nuclear and electronic stopping the two avenues in which dissipation is usually treated. This separation between electrons and ions relies on the adiabatic approximation in which ions interact via forces derived from the instantaneous electronic ground state. In a more detailed view, in which non-adiabatic effects are explicitly considered, electronic excitations alter the atomic bonding, which translates into changes in the interatomic forces. In this work, we use time dependent density functional theory and forces derived from the equations of Ehrenfest dynamics that depend instantaneously on the time-dependent electronic density. With them we analyze how the inter-ionic forces are affected by electronic excitations in a model of a Ni projectile interacting with a Ni target, a metallic system with strong electronic stopping and shallow core level states. We find that the electronic excitations induce substantial modifications to the inter-ionic forces, which translate into nuclear stopping power well above the adiabatic prediction. In particular, we observe that most of the alteration of the adiabatic potential in early times comes from the ionization of the core levels of the target ions, not readily screened by the valence electrons.

## Introduction

The interaction of energetic ions with solids is traditionally described in terms of ion-ion and ion-electron interactions, treated as decoupled phenomena. In this framework, the projectile energy loss is described in terms of nuclear, *S*_n_, and electronic, *S*_e_, stopping power. If a potential energy function between ions, *U*_binary_(**R**_*i*,*j*_), is known (*e*.*g*. from the ground state electronic energy), *S*_n_ can be calculated within classical mechanics using scattering theory in the binary collision approximation; for a detailed discussion see G. Was^1^. For the calculation of *S*_e_, the literature is abundant, covering research in the subject since the formulation of quantum mechanics, to these days. For a summary of different theories see Arista *et al*.^2^, and for practical tables of stopping see Ziegler *et al*.^3, 4^.

For ions beyond the binary collisions approximation, a view of the ion-ion interactions that includes many body effects is obtained using classical molecular dynamics, MD, to solve the problem of projectile and target atoms together, requiring a potential energy surface, PES, for the ensemble of atomic coordinates, *U*({**R**_*i*_}). The use of a PES derived from electrons in their ground state, as customarily done, is justified in terms of the Born-Oppenheimer (or adiabatic) approximation (BOA), which decouples the dynamics of fast electrons from slow moving ions^5^. However, the electronic excitations produced by swift projectiles take the system away from the ground state PES, thereby altering the forces: the actual material’s response is non-adiabatic, *i*.*e*. beyond the BOA.

Atomic scale computer simulation studies of radiation damage provide the most detailed picture of this process, with information usually much richer than that accessible experimentally. Two avenues, the classical and the quantum mechanical approaches, group the work in this area. The state-of-the-art in classical computer simulations of ion-solid interaction within the non-adiabatic picture, *i*.*e*. the process by which the perfect crystalline structure is altered by simultaneous energy deposition into the ions and the electrons, is the use of MD with empirical potentials for the ions, and the continuum heat diffusion equation for the electrons, both systems connected via electron-ion coupling terms^6^. At a higher level of complexity aiming at capturing the effects of electronic excitations but still in the realm of classical mechanics, the empirical potential in which the ions move may have a dependence on the electronic temperature^7^. Within a quantum mechanical framework, ions moving in a ‘hot’ electron bath have been described by other authors, *e*.*g*. ref. 8.

In the non-adiabatic pictures mentioned above, it is assumed that the energy deposited by the projectile into the electronic system instantaneously transforms into electronic thermal energy, *i*.*e*. heat. While this is an accurate assumption for sufficiently long times, longer than the thermalization time within the electronic system, it is not well justified at shorter times. Support to the assumption of an instantaneous thermal state for the electrons was given by Race *et al*.^9^, who studied ion-solid interactions using time-dependent tight binding theory of a metal, TD-TBT. They identified the excited electronic state as a thermal state via a comparison between the instantaneous occupation function *f* (*E*, *t*) and the Fermi function at temperature *T*, *f*_Fermi_(*E*, *T*), obtaining *T* via fitting. For a more fundamental treatment, see ref. 10.

The energy given to target electrons by energetic projectiles ultimately transforms into heat in both, ionic and electronic systems, and at a later stage these two subsystems reach mutual thermal equilibrium. In this work we address details of the early stage processes within the electronic system, before it reaches a thermal state, because in these details lies one of the most important consequences of radiation damage, namely, the production of crystalline defects. Among the processes of electronic excitation that are not thermal, we identify i- net momentum transfer to electrons and ii- ionization of the atoms in the target. Both represent energy dissipation channels that differ from heat transport, and may have consequences on the actual dynamics of the coupled ion-electron system.

In a recent work^11^ we showed that as a swift projectile passes through a solid target the momentum transfer to the target ions calculated in a non-adiabatic framework can be significantly larger than the adiabatic prediction. We concluded that it was a consequence of the electronic excitation, revealing a nontrivial connection between electronic and nuclear stopping, which is absent in the adiabatic case. These results unveiled new effects in the early stages of radiation damage cascades, which constitute the core of the present study.

In this work we use time dependent density functional theory, TD-DFT, in the pseudopotential approximation, and forces derived from Ehrenfest dynamics to analyze the ionic forces as affected by electronic excitations in a model of a Ni projectile interacting with a Ni target. Previous work by Pruneda *et al*. on non-adiabatic dynamics in insulators^12^, and by ourselves on H in Al^11, 13^, and on Ni in Ni^14^, proved that TD-DFT gives accurate results for *S*_e_ for light projectiles at high velocities *v*. Velocities are considered high or low with respect to the velocity at which the maximum in stopping occurs; for Ni this velocity is 9 a.u. of velocity. By accurate we mean in good agreement with the SRIM database, considered the standard reference for this property^15, 16^.

We choose a pseudopotential description of Ni as projectile and target because Ni has relatively shallow 2*p* core states that can be studied at moderate computational cost. Not including deeper core levels limits the quantitative accuracy of our results at high velocities, where ionization of deeper core states becomes relevant^17, 18^. Systematic investigation of the effects of the pseudopotentials on *S*_e_ is the subject of a future publication.

Electronic energy decaying into ions by phonon creation is a process absent in TD-DFT and Ehrenfest dynamics; in this theory electrons and ions do not reach thermal equilibrium, rather ionic kinetic energy is transferred to electrons until all ions are at rest^10, 19–21^. However these specific limitations of the theory are not relevant for our study because actual ion-electron thermalization occurs in time scales larger than those considered here; in fact the time scales explored in this study are so short that we do not allow target atoms to move during the simulations.

## Results and Discussion

### Stopping power

We run three types of simulations, the first type is for Ni projectiles in jellium, a uniform electron gas at a density corresponding to 10 valence electrons per Ni; the second type is for Ni projectiles moving along the center of a 〈100〉 channel in a small crystal containing 108 Ni atoms at the perfect lattice position of a fcc lattice, *i*.*e*. at time-dependent positions (*vt*, *a*_0_/4, *a*_0_/4), where *v* is the velocity (forced to be constant), *t* is time, and *a*_0_ is the lattice parameter of fcc Ni conventional unit cell; the third type of simulations has the projectile moving along the same 〈100〉 channel at position (*vt*, *a*_0_/8, *a*_0_/8), that we call off-center. At *t* = 0 the projectile is at *x* = 0 and the ground state wavefunction and energy are determined. Then, the projectile is given a velocity ν that is kept constant during the simulation. We note that, since both projectile velocities and interaction times studied here are fast and short respectively compared to target atoms dynamics, we keep target atoms at rest in all simulations, *i*.*e*. the only moving atom is the projectile. We monitor the time evolution of the total energy of the electronic system. For technical details of these simulations, see Section Simulation Methods.

**Figure 1:**
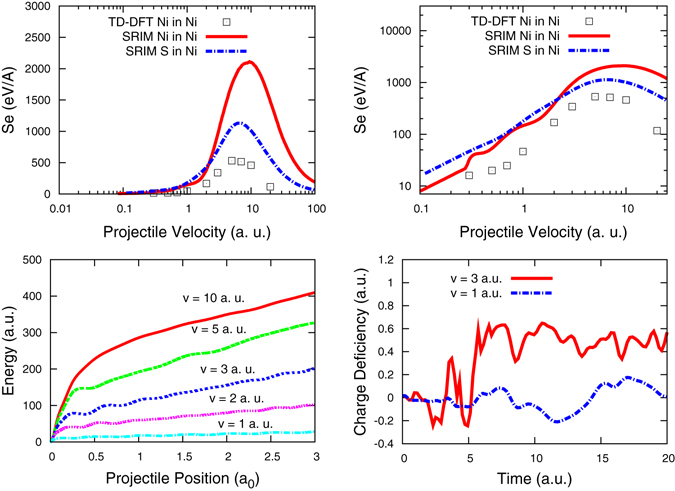
(**a**) Electronic stopping *S*_e_ of a Ni projectile traveling across a Ni target obtained using TD-DFT as described in the text (open squares); SRIM data for the same property (solid line); SRIM data for S in Ni (dash-dot line); S has the same nuclear charge than the Ni pseudoatom used in the calculations. (**b**) Same data in a log-log representation. For a discussion of similarities and discrepancies, see text. (**c**) Total electronic energy with respect to the ground state vs projectile position, for different projectile velocities. (**d**) Time evolution of the charge deficiency determined by the Bader analysis on a Ni target atom closest to the projectile trajectory.

Figure 1a shows the calculated *S*_e_ at all velocities studied, and the SRIM database for the cases of Ni into Ni and S into Ni; Fig. 1b shows the same data in log-log representation. While there is an overall agreement on the shape of this curve, with a correct location of the maximum, clearly our results do not agree with those in the SRIM database for Ni. Curiously, the agreement does not seem to improve at low velocities, where the limitations of the pesudopotential and those of the TD-DFT formalism, among them the use of an adiabatic local density approximation, should become less relevant. Figure 1c shows total electronic energy with respect to the ground state as a function of projectile position, for different projectile velocities. After an initial transient, the energy in the system increases monotonously. Note that, even though channeling projectiles travel through the host without approaching the host atoms too closely, the interaction of the projectile in the close vicinity of the host atoms is clearly seen in Fig. 1c by the oscillations that result from the periodic lattice. We determine *S*e from the average slope of these curves after the transient. Finally, Fig. 1d shows the evolution of the charge deficiency on a target atom closest to the projectile path, which we discuss below. Ionization effects are clearly seen for the case of *v* = 3 a.u. This ionized state, a deficiency of approximately 0.6 electron, is a 3*p* core hole that is not neutralized during the simulation time. We remind here that charge on an atom in a solid is an ill-defined quantity and that we use the Bader geometric algorithm to determine it^22^, as described in Section Simulation Methods.

There are multiple reasons that help explaining the discrepancies in *S*_e_ between TD-DFT results and SRIM prediction, reflected in Fig. 1a,b; among them, we identify the following:

The first is the use of a central channel trajectory in the calculation of *S*_e_. The influence of the trajectory on the value of *S*_*e*_ and its comparison with experimental results, which average all possible trajectories, has been discussed previously^13^. There it was shown that accurate values of *S*_*e*_ for protons and He in Al can be obtained if random directions, *i*.*e*. directions incommensurate with the lattice periodicity, are used in order to explore a representative ensemble of impact parameters; for velocities higher  than that corresponding to the maximum of stopping, differences of the order of 30–50% are observed. We do not explore here other trajectories in a search of better agreement, because those trajectories may involve overlap of the pseudo-atom cores (the radius of the pseudopotential we use is *r*c = 1.1 a.u.), which will add other sources of inaccuracy.

Second, it has to be noted that TD-DFT calculation of *S*_e_ for heavy nuclei using pseudopotentials can only be accurate in the low velocity limit, well below the maximum in dissipation, because in this limit ionization of the projectile electrons beyond those included in the pseudopotential can be neglected. Beyond this limit, the projectile cannot loose more charge than that explicitly included in the calculation and mimics, in our case, a pseudo Sulfur nucleus with charge *Z* = 16 and a finite size. We also reported in Fig. 1a,b the SRIM electronic stopping of S into Ni as a qualitative way to assess how close our data is to that curve; we observe that the stopping we determine close to the maximum is quantitatively similar to the S data. For even higher velocities, when also the target atoms become highly ionized beyond the 3*p* levels, our method becomes quantitatively inaccurate. The dependence of *S*_e_ on the number of core levels included has been addressed in a recent publication^13^, which shows that again, for velocities higher than that corresponding to the maximum of stopping power, differences of the order of 30–50% are observed.

Third, the SRIM database is usually assumed to be the accurate reference for stopping power. However, an interesting recent article by Wittmaack reviews the way these values are obtained and warns about their validity^23^. The SRIM *S*_*e*_ values are produced by bringing together a limited number of available experimental results in the form of ratios with respect to He stopping, *r*(*Z*_1_, He, *v*) = *S*_e_(*Z*_1_, *Z*_2_, *v*)/*S*_e_(He, *Z*_2_, *v*). *Z*_1_ and *Z*_2_ denote the atomic numbers of projectiles (with velocity *v*) and target atoms, respectively. It means that the stopping of a projectile *Z*_1_ into a target *Z*_2_ is obtained via the knowledge of the stopping of *Z*_1_ in He and the stopping of He in *Z*_2_, with no need of actual data of stopping of *Z*_1_ into *Z*_2_. Additionally, data for He impact are available in reasonable volume only for about 15% of all solid targets; missing information is derived by interpolation. In Wittmaack’s words, the electronic stopping cross sections by SRIM for velocities below the maximum of stopping are of unpredictable value and often strongly misleading because only 2 × 92 master sets of electronic stopping cross sections were required to generate all conceivable 89 × 92 tables (the tables for H, He and Li projectiles are derived separately). Additionally, several recent papers addressing stopping power at low velocities using TD-DFT conclude, that SRIM data at such velocities is inaccurate when band structure effects appear^24, 25^. Finally, the strongest argument supporting the validity of our data comes from SRIM data itself, which shows that there is no experimental information of *S*_*e*_ for Ni in Ni, and that the available data, for Ni in Cu and in Ag, for velocities below ~1 a.u. is substantially below the theoretical SRIM prediction^26^.

The arguments above, while not answering why our results differ by up to a factor of four with SRIM data, strongly suggest that low velocity stopping data is, in general, not accurately known and even more, they suggest that TD-DFT may help reduce these uncertainties, as it did in the case of light projectiles^24, 27, 28^.

Our results at low velocity do not have experimental data nor alternative theoretical predictions to be compared with. At high velocities, close to the maximum of stopping and beyond, our results are not quantitatively correct, mostly due to the first and second arguments discussed above.

We use this model despite its limitations, to provide a picture of how non-adiabatic effects modify forces on the target atoms, and to describe the ways projectile energy is deposited in the target. We believe that, while not quantitatively accurate for Ni in Ni at high velocities, the general picture and main conclusions are not altered by these limitations.

### Density, forces, and ionization charges

**Figure 2:**
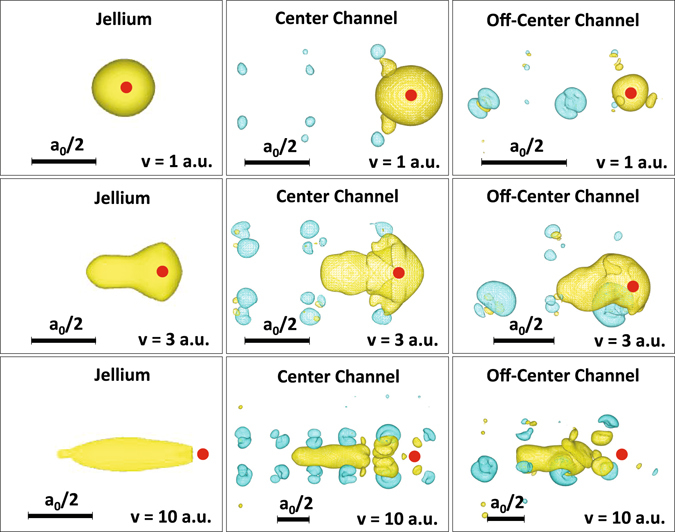
Orthogonal projections of iso-density curves showing instantaneous difference in electronic density between a Ni projectile traveling in (i) jellium at Ni metallic density (left column), (ii) along the center of a 〈100〉 channel at position (*vt*, *a*_0_/4, *a*_0_/4) (central column), and (iii) off-center along a 〈100〉 channel at position (*vt*, *a*_0_/8, *a*_0_/8) (right column) of a fcc Ni crystal, and the crystal in its electronic ground state (*a*_0_ = 3.52 Å is the Ni lattice parameter). Rows show three different projectile velocities *v*, namely 1, 3, and 10 atomic units of velocity. Yellow surfaces correspond to positive and blue to negative changes in electronic density, chosen arbitrarily at ±0.114 *e*/Å^3^. Clearly observable in all cases is the distortion of the electronic cloud of the moving projectile and, in the crystal, the ionization of those target atoms closest to the projectile path. Snapshots are taken at times after the transients observed in Fig. 1c. Solid red dot indicates the projectile position.

Figure 2 shows snapshots of orthogonal projections of iso-density electronic surfaces for a projectile in jellium (left column), center channel (center column), and off-center channel (right column) trajectories as defined in the previous Section, at 1, 3, and 10 a.u. of velocity.

As the projectile moves along its path in jellium, an electronic charge tail develops. As the projectile velocity increases, the tail length increases, showing in a graphical way the projectile ionization. These density perturbations are similar to those studied by Echenique *et al*. in jellium using Lindhard response theory^29, 30^.

A similar electronic charge tail is observed in snapshots corresponding to the projectile traveling in the Ni target, for both the center and off-center channeling trajectories. Projectile electrons lag behind the projectile nucleus, but the presence of the target nuclei significantly distorts the electronic charge distribution. In particular, we observe that target atoms along the projectile track get stripped from their electrons creating semi-core holes and becoming positively charged (blue iso-surfaces), suggesting the possibility of a strong Coulomb repulsion between target nuclei. Finally, the projectile tail sitting between two ionized target atoms across the channel also suggests the possibility of shielding the Coulomb repulsion between ionized target atoms, and providing eventually a net attraction for a short period of time right after the projectile passes by.

**Figure 3:**
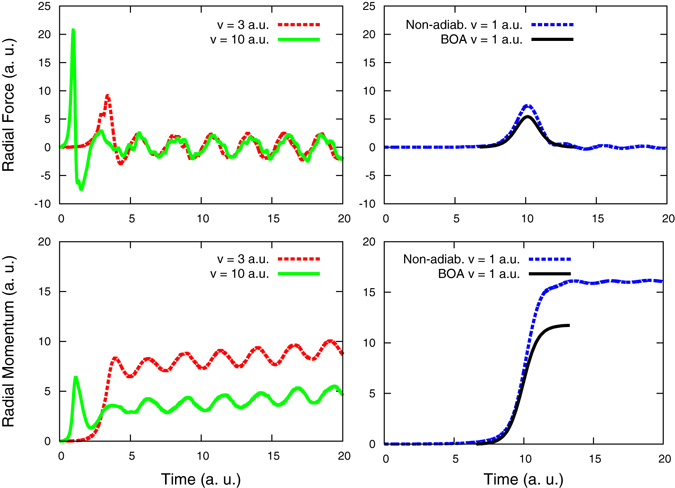
Non-adiabatic radial forces and radial momenta vs time on a target atom closest to the projectile path for a projectile traversing the sample at the center of a 〈100〉 channel; Fig. 3a,c for velocities *v* = 3 and 10 a.u., and Fig. 3b,d for velocity 1 a.u. Fig. 3b,d also show force and momentum for the adiabatic approximation BOA at *v* = 1 a.u.

Figure 3a,b show the time dependence of the non-adiabatic radial forces on a Ni target atom closest to the center of the channel, at a radial distance of $\sqrt{2}/4$ *a*_0_ from the axis defined by the projectile trajectory, for velocities *v* = 1, 3, and 10 a.u. Figure 3b also shows the result of the adiabatic BOA, *i*.*e*. the force obtained from the instantaneous ground state electronic energy; an ordinary time-independent DFT calculation is performed to obtain the converged ground state results that are required to compute these forces. The magnitude of the BOA force depends only on projectile-target atom relative position, *i*.*e*. **F** = **F**(*r*_*rel*_), independent of the projectile velocity for high projectile velocity (high projectile velocity implies the assumption that target atoms do not have time to move significantly during the interaction time). Therefore the BOA force at other velocities can be obtained by re-scaling the time-axis.

In all four cases, as the projectile approaches a target atom, a positive (outwards) radial force appears that reaches its maximum value for the closest approximation. At large projectile velocities (*v* = 3 and 10 a.u.), a surprising negative (inwards) force develops right after the maximum, suggesting that nuclei attract each other, as qualitatively implied by the electronic density distribution reported in Fig. 2. After these features, periodic oscillations appear linked to plasmons excited in the system; they persist due to the small box size and the use of periodic boundary conditions. They do not average to zero, but to a positive value indicating a remnant radial repulsive force.

Figure 3c,d, show the time integral of the non-adiabatic force, which represents the radial momentum transfer ${\rm{\Delta }}{\bf{P}}=\int {\bf{F}}{\rm{d}}t$ by the projectile to the target atoms. The fact that for velocities *v* = 3 and 10 a.u. the force does not return to zero translates into a monotonous increase in momentum transfer for as long as the force is not zero. For *v* = 1 a.u. this effect is negligible. The remnant radial repulsive force can be determined from the average slope of the curves in Fig. 3c, which can be described by Δ*P* = *a* + *bt* at long times, where *t* is time. For example, for *v* = 3 a.u., *a* = 6.96 and *b* = 0.116, both in a.u.

The momentum transfer in the BOA case, Fig. 3d, occurs during the interaction time and ceases to increase after the projectile has passed because the force returns to zero. It gives a radial momentum transfer from the projectile to the target atom proportional to 1/*v*, which is the standard result, since ${\rm{\Delta }}{\bf{P}}=\int {\bf{F}}({r}_{rel}){\rm{d}}t=1/v\int {\bf{F}}({r}_{rel}){\rm{dr}}$. For a detailed discussion of this limit see Chapter 1, in the book by G. Was^1^.

Momentum, or energy, transfer to the target ions is what the nuclear stopping power, *S*_n_, accounts for; Fig. 3 shows that when forces between ions are calculated in a non-adiabatic framework, *S*_n_ is larger than the adiabatic prediction.

From Figs 2 and 3 we conclude that as the projectile passes by, target nuclei are partially stripped off their electrons, increasing the maximum strength of the repulsive peak force with respect to the adiabatic case, then attract each other for a short time, presumably due to screening/bonding by the projectile electronic tail, and later repel each other with a remnant radial repulsive force, presumably via Coulomb repulsion between ionized target atoms.

**Figure 4:**
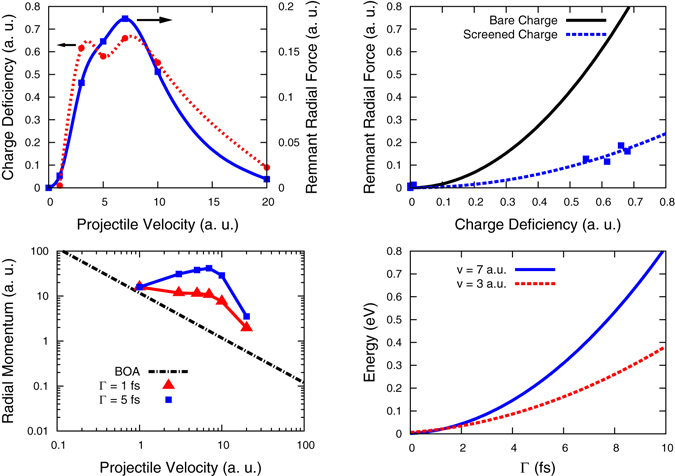
(**a**) Charge deficiency and remnant radial force vs projectile velocity on an atom nearest to the projectile trajectory at the center of the channel. These values are taken long after the projectile has traveled across the sample. (**b**) Squares: Remnant radial force on an atom closest to the projectile trajectory as a function of charge deficiency. Dash-dot line: fit of TD-DFT data, ${F}_{r}^{actual}$ = 0.37 *q* ^2^. Solid line: Bare Coulomb radial force for atoms at the same position (see text) ${F}_{r}^{bare}$ = 1.7 *q* ^2^. (**c**) Radial momentum transferred to atoms closest to the projectile path vs projectile velocity. Dash-dot line corresponds to the BOA and is proportional to 1/*v*. Triangles (squares) correspond to momentum transferred under the assumption that the lifetime of the core hole, Γ, is 1 (5) fs. (**d**) Energy transferred to the target atoms nearest to the track vs lifetime of the core hole, for projectile velocities *v* = 3 and 7 a.u.

To explore the relation between the remnant radial repulsive force and the charge of the ionized target atoms, we show in Fig. 4a both quantities as a function of projectile velocity; they clearly seem correlated. The maximum radial force corresponds to the maximum of ionization, and it occurs for projectile velocity *v* $\simeq$ 7 a.u., or an energy of ~71 MeV, which corresponds closely to the maximum in stopping power as given both by SRIM^15, 16^ and by our calculations, see Fig. 1a. The similarity of these curves strongly suggests that the origin of the non-adiabatic component of the radial forces is the Coulomb repulsion between the ionized atoms.

One way to analyze the origin of these forces is to compare their strength with the Coulomb forces corresponding to a system of bare charges at the same location of the atoms in the channel, which would give an upper bound. Since our results show significant charge only for those atoms nearest to the channel, and because metallic screening lowers the effect of distant atoms, we only consider the five closest atoms acting on each channel atom for the calculation of the remnant force. These atoms are themselves channel atoms and bare the same charge. If the atom under consideration for the force calculation is at the origin (0, 0, 0), and the trajectory is defined by a projectile position (*vt*, *a*_0_/4, *a*_0_/4), then the five atoms are nearest neighbors located at (0, *a*_0_/2, *a*_0_/2), (±*a*_0_/2, *a*_0_/2, 0), (±*a*_0_/2, 0, *a*_0_/2). The resulting radial force for bare charges *q* in vacuum is ${F}_{r}^{bare}$ = 1.7 *q* ^2^ in a.u., where the 1.7 is a geometric factor determined by the positions of the five atoms defined above. This function is shown in Fig. 4b. This figure also shows the actual relation between charge and force, obtained from Fig. 4a, which is best fitted by ${F}_{r}^{actual}$ = 0.37 *q* ^2^. The similarity supports the claim that remnant forces come from ionization of core electrons.

Metallic screening is responsible for a force smaller than that corresponding to bare charges: an effective charge of 0.47 *q* accounts for the force, since ${F}_{r}^{actual}$ = 1.7 × (0.47 *q*)^2^. Screening by conduction electrons at these short time scales is weaker than the static screening, as can be estimated from the fact that for Ni, with 10 electrons per atom, *r*_s_ = 1.23 a.u. (*r*_s_ is the radius of a sphere containing one electron, an alternative measure of density), and using the static Thomas Fermi theory, the corresponding screening length is λ = 0.72 a.u., implying that the effective charge under static metallic screening seen at the distances under consideration becomes 0.0015 *q*, negligible compared to the 0.47 *q* that we obtain. We note that screening is not the only possible source of effective charge reduction, because the charges we determine with the Bader scheme are only approximate.

We conclude that ionization of core electrons is the main source of the modification of the atomic forces with respect to the adiabatic counterpart. This ionization is ineffectively screened by conduction electrons at the the short time scales involved here.

The momentum transfer to the target atoms increases linearly with time during the lifetime of the core hole, Γ, as Fig. 3c suggests. Core holes are filled by either a radiative decay of a valence electron, or by Auger two-electron process, both mechanisms absent in TD-DFT^31^. Therefore our computational technique cannot  predict lifetimes. Even if it could, it would be beyond the time scale TD-DFT can reach. We resort to values for Γ from the literature.

Ohno *et al*.^32^ report experimental and theoretical hole lifetimes for most atomic elements in the periodic table, which show considerable dispersion. From this atomic data, we estimate the lifetime of a 3*p* hole in crystalline Ni to be in the range of 0.5–5 fs.

In Fig. 4c we report the total momentum transfer for two representative values of the 3*p* core hole lifetime Γ in Ni, namely 1 and 5 fs. We observe that the total momentum transfer could be significantly greater than the adiabatic result when non-adiabatic effects are included in the calculation of the interatomic forces. For comparison, Fig. 4c also shows the adiabatic BOA result. This observation can be rephrased in terms of *S*_n_, namely: the nuclear stopping power *S*_n_ that results from considering non-adiabatic effects could be considerably greater that the adiabatic result.

We discuss now the effect of this momentum transfer on the energy deposition mechanisms in the crystal. If we assume the target atoms are at rest before being ionized (thermal velocities for Ni are in the range of 10^−4^ a.u. and can therefore be neglected), we can estimate the energy transferred to them as (Δ*P*)^2^/2 *m*_*Ni*_; a plot of this energy transfer as a function of core-hole lifetime Γ is shown in Fig. 4d, which indicates that the energy transfer is in the range of a sizable fraction of an eV.

Summarizing, due to ionization of metal atoms along a track, energy in the range of a fraction of an eV is transferred to those atoms in the form of an outward radial momentum. It represents a significant increase in momentum transfer with respect to the adiabatic nuclear stopping power, as shown in Fig. 4c.

## Conclusion

Using non-adiabatic TD-DFT, we showed details of the energy deposition mechanisms associated to the passage of energetic ions in a metallic target. Similar to the Coulomb explosion in insulators, although much weaker, our work shows that in metals, momentum is transferred to the target atoms due to ionization effects right after the projectile passes. This effect creates an additional contribution to the projectile energy deposition to ions that is beyond that originated by an adiabatic projectile-ion potential (the traditional nuclear stopping power, *S*_n_). These results provide a semi-quantitative explanation to the observation we reported previously^11^.

This energy deposition has the form of a collective radial outward excitation in the time scale of a few fs after the projectile passage. While this energy is below the threshold for permanent atomic displacement (*i*.*e*. energy needed to create a Frenkel pair in an otherwise perfect lattice, which is in the order of few tens of eV for most metals), it represents a collective excitation with cylindrical symmetry that generates an outgoing pressure wave.

We note that our results are quantitatively accurate only in the limit of low velocities, well below ~9 a.u. where the maximum in dissipation occurs. At lower velocities, ionization of core levels is incipient and our treatment of only 3*p* core electrons is sound. At velocities larger than that, our pseudopotential approach prevents the projectile and the target to ionize beyond 16 electrons. In a more realistic calculation, ionization charges, forces, and transfer of momentum and energy, may all be much larger, implying that the effect we report here, *i*.*e*. a collective radial-out force on the atoms close to the channel due to Coulomb repulsion, may be much more important than our estimate. We believe that the fact that our model is not quantitatively accurate for the case of Ni in Ni at high velocity, does not invalidate the general conclusion that a contribution to the nuclear stopping power *S*_n_ comes from the electronic excitations, making it substantially larger than the adiabatic prediction.

## Simulation Methods

For this study, we use TD-DFT^33^ that describes the relevant parts of the physics involved in this problem, despite the numerous approximations of practical implementations. TD-DFT is a quantum mechanical theory used to investigate the dynamics of many-body systems, in particular in the presence of time-dependent potentials. The method we use to calculate electronic stopping in this work follows that of Pruneda *et al*.^12^ and consists of a discrete time-integration of the time-dependent Kohn-Sham (TD-KS) equations for the one-electron orbitals *ϕ*_*i*_(**r**, *t*)^34, 35^,

1 $$i\hslash \frac{\partial {\varphi }_{i}({\bf{r}},t)}{\partial t}=\{-\frac{{\hslash }^{2}{\nabla }^{2}}{2m}+{\hat{V}}_{{\rm{ext}}}\,(\{{R}_{I}\})+{\hat{V}}_{{\rm{HXC}}}\,[n({\bf{r}},t)]\}\,{\varphi }_{i}\,({\bf{r}},t)$$

In Eq. 1, **r** is the spatial coordinate and *t* is time. ${\hat{V}}_{{\rm{HXC}}}$[*n*(**r**, *t*)] describes both the electrostatic (Hartree) electron-electron interaction and the quantum-mechanical exchange-correlation (XC) potential for which we use the adiabatic local-density approximation (ALDA)^36, 37^; *n*(**r**, *t*) is the electron density. The time-dependent potential acting on the electrons, ${\hat{V}}_{ext}$(*t*), is given by norm-conserving pseudopotentials representing the ionic system, which take the form of a local, atom centered, smooth potential plus a separable, Kleinman-Bylander non-local operator, as described in ref. 38; its time dependence arises from both the fast-moving projectile and the motion of the target atoms, although in this work, all target atoms are kept at rest because in the short time scale and the type of channeling trajectories we study, they do not move significantly.

The model is completed by the equations for the force on the nuclei in Ehrenfest dynamics,

2 $${{\bf{F}}}_{I}(t)=-\sum _{i}\langle {\varphi }_{i}(t)|{{\boldsymbol{\nabla }}}_{{R}_{I}}{\hat{H}}_{{\rm{e}}}|{\varphi }_{i}(t)\rangle $$

where the gradient is taken at the position of atom *I*, ***R***_*I*_ (*t*), and ${\hat{H}}_{{\rm{e}}}$ is the operator in the r.h.s. of Eq. 1.

Integration of Eq. 1 gives time dependent electronic wave functions and density, and Eq. 2 gives the forces used in this work to calculate momentum and energy. Following our own convergence tests and the literature^39^, the fourth-order Runge-Kutta scheme used to propagate the electronic orbitals in time shows numerical stability for a time step of 0.121 attoseconds for the highest Ni velocity explored, 10 a.u.; we have used this value for all velocities.

Time propagation of the electronic wavefunction requires an initial condition (KS states and electron density), which depends on the target nuclei and projectile positions. We note that both projectile velocities and interaction times studied here are fast and short respectively compared to target atoms dynamics. Therefore during the time span in which the projectile -target atom force acts, target atoms move a negligibly small distance; the momentum transfer acts in practice as an initial condition for subsequent motion. For that reason we keep the target atoms at rest in their equilibrium fcc positions in all simulations, *i*.*e*. the only moving atom is the projectile, which is forced to move at constant velocity. These conditions simplify the analysis.

We place the projectile at rest at the center of a 〈100〉 channel, and the initial electronic state is obtained by solving the ground state (time-independent) KS equations for the target plus projectile system.

To compute the electronic stopping, we use the time-dependent energy *E*(*t*),

3 $$E(t)=\sum _{i}\int {\rm{d}}{\bf{r}}{\varphi }_{i}^{\ast }({\bf{r}},t)\,[-\tfrac{{\hslash }^{2}{\nabla }^{2}}{2m}+{\hat{V}}_{{\rm{ext}}}(t)]\,{\varphi }_{i}\,({\bf{r}},t)+{E}_{{\rm{HXC}}}\,[n({\bf{r}},t)]+{E}_{\text{ion}{\rm{-}}\text{ion}}(t)$$

which comprises the electronic kinetic energy, the external (electron-ion) potential ${\hat{V}}_{{\rm{ext}}}$(*t*), the electron-electron interaction *E*_HXC_[*n*(*t*)] (Hartree interaction and XC density-functional approximation), and the configurational energy *E*_ion-ion_(*t*). The external potential is incorporated in the pseudopotential approximation with consideration of 16 explicit electrons (3*p*, 4*s*, and 3*d* electrons). *E*(*t*) reported here is the total energy of the system without the kinetic energy of the ions (projectile and target ions). The rate of increase of *E*(*t*) as a function of time or projectile displacement *x*(*t*) gives the instantaneous electronic stopping power *S*_e_ via,

4 $${S}_{{\rm{e}}}\,(x)=-{\rm{d}}E(t)/{\rm{d}}x(t)$$

It is important to stress the point that TD-DFT is an *ab initio* theory in the sense that there are no adjustable parameters, once the basic approximations as described above have been defined and adopted. This approach does not use any *a priori* screening model or a predefined ionized state. Screening and ionization are controlled by the response according to TD-DFT equations. We note that more sophisticated approaches including exact many-body and dynamic exchange-correlation treatments are available in the literature^40, 41^.

To represent the target we use a supercell with 108 Ni atoms on a fcc 3 × 3 × 3 conventional lattice cells, with a lattice parameter *a*_0_ = 3.52 Å. Boundary conditions are periodic in the 3 spatial directions for the density and also for the wavefunction (Γ-only sampling). The initial condition is the ground state of the system composed of these 108 Ni, and an additional, interstitial, Ni atom, represented by the same pseudopotential than those in the fcc crystal lattice, which will become the projectile when velocity is imposed to it. Important to highlight, the 109-atom sample is electrically neutral during the entire simulation, a situation different from linear response approaches to electronic stopping, where the charge state of the projectile needs to be specified *a priori*; here the charge state of the projectile as well as the dynamic screening by the target electrons are the output, the result of the TD-DFT self-consistent solution of the combined target-plus-projectile system.

A norm-conserving pseudopotential with a cutoff radius of *r*_*c*_ = 1.1 a.u. and an energy cutoff of 150 *Ry* for a plane wave basis are used. This cutoff value is chosen after calibration runs, and proves to be high enough for our purposes. The calculations include 10 valence electrons (4*s* and 3*d*) and 6 semi-core electrons (3*p* states) per atom (*i*.*e*. there are 1774 electrons in the system), and are nonmagnetic. Simulations extend over time lengths up to *t* ~ 100 a.u, or 2.4 fs. For details on the implementation of TD-DFT in QBOX, see ref. 39, and for a discussion about the influence of the finite pseudopotential radius on calculations of stopping, see ref. 13.

We run three types of simulations, the first type is for projectiles in jellium, a uniform electron gas at a density corresponding to 10 valence electrons per Ni atom, *i*.*e*. 1080 electrons in the same 3 × 3 × 3 *a*_0_ box obtained by removing the pseudopotentials associated to the Ni target nuclei; the second type is for projectiles moving along the center of a 〈100〉 channel of the fcc lattice, *i*.*e*. at time-dependent positions (*vt*, *a*_0_/4, *a*_0_/4), where *v* is the velocity (forced to be constant), *t* is time, and *a*_0_ is the lattice parameter of fcc Ni conventional unit cell, and the third type of simulations has the projectiles moving along the same 〈100〉 channel at position (*vt*, *a*_0_/8, *a*_0_/8), that we call off-center. At *t* = 0 the projectile is at *x* = 0 and the ground state wavefunction and energy are determined.

With this initial state, a fix velocity is imposed to the projectile (*v* = 0.3, 0.5, 0.7, 1, 2, 3, 5, 7, and 10 a.u. of velocity, corresponding to kinetic energies *K* = 0.13, 0.36, 0.71, 1.5, 5.8, 13, 36, 71, and 146 MeV). When the projectile travels a distance equal to the cell size, *i*.*e*. three lattice cells or 19.96 a.u. of distance, it re-enters the simulation box due to the periodic boundary conditions. At this moment, the velocity is reset to zero and the TD-DFT simulation continues with purely electron dynamics since all ions are now stationary. These two sudden perturbations (setting the projectile in motion and stopping it) produce transients that have a relatively short lifetime and are clear in the behavior of the energy versus position; relevant results are obtained from the stationary state away from those transients. To minimize its effects, we measure forces and charges on an atom in the center of the supercell, the farthest away from the location of the perturbations.

Charge analysis is performed using Bader charge analysis algorithm^22^. For a given electronic charge density, the Bader partitioning scheme is defined by the contour of minimal gradient around each atom, providing a measure of the atomic charge, and a quantitative determination of the relative degree of ionization. Electronic charge density differences are visualized using the 3-D software VESTA^42^. In all cases, charge density differences are taken between the configuration in the dynamical simulation and the ground state of the 108-atom crystal, without the projectile.
